# Editorial: July 2022: sarcoma awareness month

**DOI:** 10.3389/fendo.2024.1480176

**Published:** 2024-08-30

**Authors:** Liam Chen

**Affiliations:** Department of Laboratory Medicine and Pathology, University of Minnesota Medical School, Minneapolis, MN, United States

**Keywords:** sarcoma, soft tissue, bone, muscle, endocrine, diagnostics

Sarcomas is the general term for rare kinds of cancer growing in the bone and connective tissues. These tumors can be divided into two main groups, soft tissue sarcomas, and bone sarcomas. Interestingly, a new kind of sarcoma has been recently described, called pseudoendocrine sarcoma (1). The most common tissues affected by this kinds of sarcoma are bones, muscles, tendons, cartilage, nerves, fat, and blood vessels as well as many endocrine organs. It affects mainly children, adolescents, and adults under the age of 30, and represents 1% to 2% of all cancers. Symptoms vary depending on the part of the body that is affected, and prompt therapies are primary for increasing the survival rate of the patients. Most of the treatments for this kinds of cancers may include chemotherapy, radiation therapy and surgery. Recently, hormonal therapy has revealed to be a great strategy for the treatment of some of these tumors, especially for uterine and endometrial stromal sarcomas.

It is in this spirit that Frontiers has launched a new article collection to coincide with the Sarcoma Awareness Month. This Frontiers in Endocrinology Research Topic entitled “July 2022: Sarcoma Awareness Month” aims to address the endocrinology-specific dimensions of this devastating disease, highlighting the importance of sarcoma research and considering how to improve treatment options and prognostic predictions.

Liu et al. explored the role of copper dysregulation in osteosarcoma (OS) by identifying cuproptosis-related long non-coding RNAs (CRLs) and their potential in prognostication and treatment response. Using differential expression and correlation analyses, a CRL signature comprising four key CRLs was developed. This signature was validated through Kaplan-Meier survival analysis, ROC curves, and independent prognostic assessments, demonstrating its ability to stratify patients into low- and high-risk groups with significant differences in prognosis. The signature also correlated with immune status, responses to immunotherapy, and chemotherapy sensitivity. The findings, confirmed by RT-qPCR, suggest that this CRL signature could enhance prognostic evaluations and guide personalized treatment strategies in OS.

Study done by Zhuang et al. aimed to develop a nomogram for predicting overall survival (OS) in patients with retroperitoneal leiomyosarcoma (RLMS) following surgical resection, as no such model existed. Analyzing 118 patients who underwent surgery between September 2010 and December 2020, the researchers constructed the nomogram using Cox regression, incorporating factors such as the number of resected organs, tumor diameter, FNCLCC grade, and multifocality of lesions. The median OS was 47.8 months, with a majority of tumors fully resected. The nomogram demonstrated good predictive performance, with a concordance index of 0.779 and strong agreement between predicted and actual OS in calibration curves. This model offers valuable guidance for postoperative consultation and patient selection for clinical trials.

Another study performed by Eichler et al. investigated the Health-Related Quality of Life (HRQoL) of adult sarcoma patients and survivors through longitudinal assessment over one year across 39 centers in Germany. Utilizing the EORTC QLQ-C30 questionnaire, researchers followed 1111 patients at baseline, with 915 continuing at 6 months and 847 at 12 months. Analysis revealed that HRQoL varied based on tumor location, with lower extremity sarcoma patients reporting poorer outcomes compared to those with upper extremity sarcomas. Additionally, treatment involving radiotherapy or systemic therapy was linked to decreased HRQoL. Among patients in complete remission, smoking negatively impacted HRQoL. Bone sarcomas consistently showed the worst HRQoL scores, and factors such as being female, aged 55-64, lower socioeconomic status, and comorbidities were associated with poorer HRQoL across both groups. Despite some improvement in HRQoL over time and with physical activity, the study highlights the need for targeted strategies to enhance HRQoL in sarcoma patients, particularly for those with bone sarcomas and other identified risk factors.

The six case reports covers interesting, yet rare sarcoma cases including hepatic inflammatory pseudotumor-like follicular dendritic cell sarcoma (Ding et al.), primary synovial sarcoma of the thyroid gland (Ren et al.), primary mediastinal Ewing’s sarcoma (Su et al.), low-grade fibromyxoid sarcoma (Zhang et al.), and two primary pulmonary sarcomas (Wen et al.; Zhang et al.). Through analyzing the distinctive clinicopathologic features of these rare cases combined with literature review, valuable insights and lessons have been learned which will help better understand and manage these diseases.

With over 70 different subtypes, sarcomas can be difficult to diagnose and treat. The month-long sarcoma awareness observance seeks to educate the public about the symptoms, risks, and the need for early detection, while also supporting research and funding for improved treatments. While highlight the challenges faced by those affected, articles collected in this topic undoubtedly would drive progress in the fight against this challenging disease.
